# 2306. A serosurvey of healthcare workers caring for, or handling specimens from, individuals exposed to, or diagnosed with, SARS-CoV-2 infection (COVID-HEROES)

**DOI:** 10.1093/ofid/ofad500.1928

**Published:** 2023-11-27

**Authors:** Alyssa Pradhan, Dominic E Dwyer, Linda Hueston, Will Asquith, Ravindra Dotel, Ralph Nanan, Nicholas Wood, Annaleise Howard-Jones, Gabrielle M O’Kane, Rhonda Stuart, Naomi Whyler, Kyra Chua, Timothy Gilbey, Timothy Bemand, Chris Weatherall, Mohamed Hammoud, Marianne Martinello, Neela Joshi Rai, Matthew O’Sullivan

**Affiliations:** Westmead Hospital, NSW, Sydney, New South Wales, Australia; University of Sydney, NSW, NSW Health Pathology - Institute of Clinical Pathology and Medical Research, Westmead Hospital, NSW, Sydney, New South Wales, Australia; NSW Health Pathology - Institute of Clinical Pathology and Medical Research, Westmead Hospital, NSW, Sydney, New South Wales, Australia; Institute of Clinical Pathology and Medical Research, Westmead Hospital, NSW, Sydney, New South Wales, Australia; Blacktown Hospital and Bankstown Hospital, Sydney, New South Wales, Australia; The Children's Hospital at Westmead, NSW, Sydney, New South Wales, Australia; The Children's Hospital at Westmead, NSW, Sydney, New South Wales, Australia; NSW Health Pathology, Sydney, Alabama; Pathology NSW, Gosford, New South Wales, Australia; Monash Heath, VIC, Monash University, VIC, Melbourne, Victoria, Australia; Monash Heath, VIC, Melbourne, Victoria, Australia; Austin Health, Heidelberg, Victoria, Australia; Wagga Wagga Base Hospital, NSW, Wagga Wagga, New South Wales, Australia; Wagga Wagga Base Hospital, NSW, Wagga Wagga, New South Wales, Australia; St George Hospital, NSW, Sydney, New South Wales, Australia; The Kirby Institute, UNSW Sydney, NSW, Sydney, New South Wales, Australia; The Kirby Institute, UNSW Sydney, NSW, Sydney, New South Wales, Australia; Centre for Infectious Diseases and Microbiology, Westmead Hospital, NSW, Sydney, New South Wales, Australia; Westmead Hospital, NSW, University of Sydney, NSW, Sydney, New South Wales, Australia

## Abstract

**Background:**

COVID-HEROES is a multicentre study of Australian healthcare workers (HCW) assessing 1) incidence of and risk factors for SARS-CoV-2 infection and 2) serological response to SARS-CoV-2 vaccination and infection.

**Figure 1:**
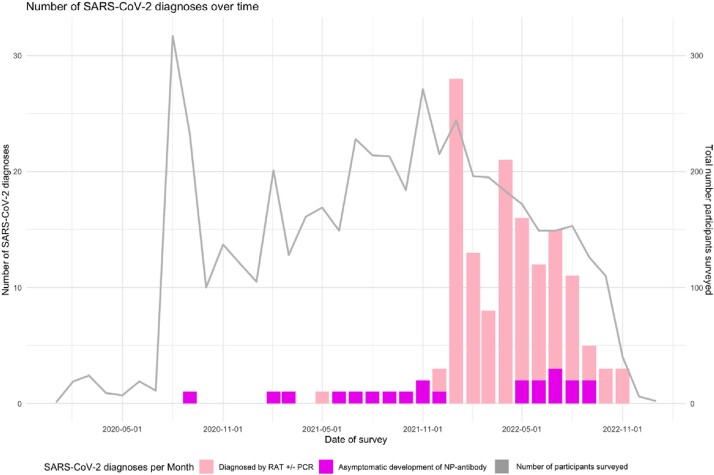
Cases of SARS-CoV-2 over time by diagnostic method

**Methods:**

We conducted a multi-centre, prospective observational cohort study of Australian HCWs from January 2020 – January 2023, evaluating incidence of, and risk factors for, SARS-CoV-2 infection. HCWs underwent monthly surveys to assess SARS-CoV-2 occupational exposure, vaccination status and infection and serology testing to detect antibody response to vaccination (measured by IgG immunofluorescent antibody titre) or infection (development of SARS-CoV-2 nucleoprotein (NP) IgG antibodies measured by ELISA). Factors associated with infection were identified using logistic regression models.

**Figure:**
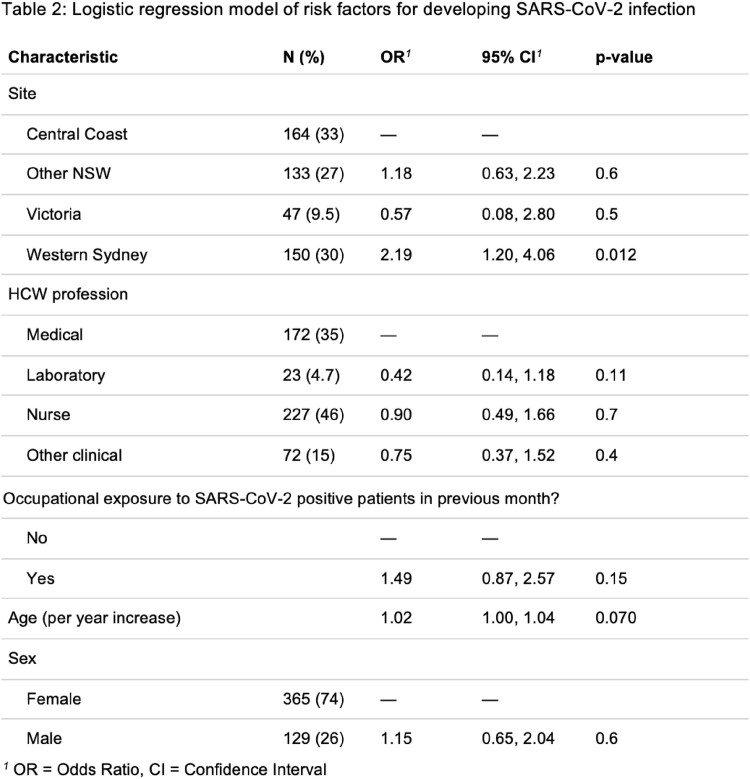

**Results:**

575 participants completed 4882 surveys and 4325 serological tests over 36 months. Median enrolment duration was 8.4 (IQR 2.2-17.4) months. 160/575 (28%) of participants reported SARS-CoV-2 infection diagnosed by polymerase chain reaction (PCR) and/or rapid antigen test (RAT). 101/160 (64%) participants with known infection developed NP antibodies. A further 41 participants, without reported infection developed NP antibodies, indicating unrecognised infection (Figure 1).

153/160 (96%) known infections occurred after December 2021, corresponding to the Omicron wave, despite 25-50% of participants reporting occupational exposure to SARS-CoV-2 patients throughout the study period. 571/575 participants developed IgG response following two doses of SARS-CoV-2 vaccine, 6 of whom seroreverted. A logistic regression model of risk factors for HCWs contracting SARS-CoV-2 infection (Table 2) did not identify significant association between occupational exposure and infection (OR 1.49, CI 0.87- 2.57, p = 0.15).

**Conclusion:**

In this 3-year prospective cohort, we report detailed occupational SARS-CoV-2 exposures to assess nosocomial acquisition risk, including symptomatic and unrecognised infection. We demonstrate low rates of infection until community prevalence increased, despite significant workplace exposure, indicating effective use of protective precautions in the Australian healthcare setting.

**Disclosures:**

**All Authors**: No reported disclosures

